# Balancing Osseointegration and Infection Control: The Role of Titanium Surface Topography in Peri-Implant Biology

**DOI:** 10.3390/jfb17070327

**Published:** 2026-07-06

**Authors:** Simina Angela Lăcrimioara Iușan, Dana-Gabriela Feștilă, Ioana-Codruța Mirică, Giorgiana Corina Mureșan, Bianca-Nausica Petrescu, Olga Sorițău, Carmen Costache, Dan-Alexandru Toc, Otilia Andercou, Maria Aluaș, Simion Bran, Dragoș Budei, Silviu Albu, Ondine Patricia Lucaciu

**Affiliations:** 1 Department of Oral Health, Iuliu Hatieganu University of Medicine and Pharmacy, 400012 Cluj-Napoca, Romania; siminaiusan@yahoo.com (S.A.L.I.); muresan_giorgiana_corina@elearn.umfcluj.ro (G.C.M.); nausicapetrescu13@gmail.com (B.-N.P.); maria.aluas@elearn.umfcluj.ro (M.A.); ondineluc@yahoo.com (O.P.L.); 2 Orthodontics Department, Faculty of Dental Medicine, Iuliu Hațieganu University of Medicine and Pharmacy, 400012 Cluj-Napoca, Romania; dana.festila@umfcluj.ro; 3 Department of Tumor Biology and Radiobiology, Oncology Institute “Prof. Dr. Ion Chiricuta”, 400015 Cluj-Napoca, Romania; olgasoritau@yahoo.com (O.S.); otiliabarbos2006@yahoo.com (O.A.); 4 Department of Microbiology, Iuliu Hatieganu University of Medicine and Pharmacy, 400347 Cluj-Napoca, Romania; carmen_costache@yahoo.com (C.C.); dan_alext@yahoo.com (D.-A.T.); 5 Department of Maxillofacial Surgery and Implantology, “Iuliu Hatieganu” University of Medicine and Pharmacy, 400012 Cluj-Napoca, Romania; dr_brans@umfcluj.ro; 6 Dentix Millennium SRL, 087153 Giurgiu, Romania; budei.dragos@dentixmillennium.ro; 7 II-nd Department of Otolaryngology, Iuliu Hatieganu University of Medicine and Pharmacy, 400012 Cluj-Napoca, Romania; silviualbu63@elearn.umfcluj.ro

**Keywords:** titanium implants, nanostructured surfaces, bacterial adhesion, biofilm formation, osseointegration, *E. faecalis*, *S. oralis*

## Abstract

Background: Peri-implant infections remain a major cause of dental implant failure, largely due to bacterial adhesion and biofilm formation on implant surfaces. This study aimed to investigate how surface topography influences bacterial colonisation and osteoblastic response. Methods: Titanium discs with machined (Ma), sandblasted, large-grit, and acid-etched (SLA), and nanostructured (Nano) surfaces were prepared, sterilised, and seeded with pre-differentiated dental follicle mesenchymal stem cells. Co-cultures with *Enterococcus faecalis (E. faecalis)* and *Streptococcus oralis (S. oralis)* were established under CO_2_-free conditions, and cell–bacteria interactions were evaluated using fluorescence microscopy and quantitative image analysis. Results: Nano surfaces showed the highest osteoblastic adhesion and viability, while significantly reducing bacterial proliferation and biofilm formation compared with Ma and SLA surfaces. The sequence of colonisation influenced cell–bacteria dynamics, with early cell attachment limiting subsequent bacterial adhesion. Conclusions: Nano titanium surfaces may offer a dual benefit by promoting osseointegration while limiting bacterial adhesion. These findings support their potential use as surface modifications to reduce peri-implant infection risk and improve long-term implant success.

## 1. Introduction

Titanium dental implants are widely used in the prosthetic rehabilitation of patients with partial or complete edentulism, owing to their excellent mechanical properties, corrosion resistance, and superior biocompatibility [1,2]. However, despite these advantages, infections associated with implants (Biomaterial-Associated Infections—BAI) represent a common and serious complication, responsible for the failure of many treatments. It is estimated that, in the United States, device-associated infections are estimated to account for over 25% of all reported nosocomial infections [3].

The placement of an implant triggers a complex host immune response, known as a foreign body reaction, which involves acute and chronic inflammation, granulation tissue formation, and fibrous encapsulation. These events may lead to the occurrence of a local area of immunosuppression (*locus minoris resistentiae*), favourable to bacterial colonisation [4,5].

The first step in the development of an infection is the adhesion of bacteria to the surface of the biomaterial, followed by the formation of a biofilm—a structure that protects microorganisms from the attack of the immune system and from antibiotics [3,6]. This biological barrier makes most implant-associated infections become chronic, often requiring the removal of the device [3].

According to the “race for the surface” theory, there is a competition between host cells and bacteria for the colonisation of the implant surface. If the host cells succeed in adhering first, a protective cellular layer is formed that reduces the risk of infection. On the other hand, if bacteria colonise first, the implant becomes a substrate for the development of the biofilm, which prevents tissue integration and increases the risk of failure [2,4,5,6].

The development of advanced biomaterials that support osseointegration but also prevent bacterial colonisation is a major focus of current biomedical research [6]. These solutions are necessary not only because of the limitations of the immune system in the presence of a foreign body, but also because of the increasing bacterial resistance to antibiotics, which makes many of the conventional prophylactic strategies ineffective [2,3].

The aim of this in vitro experimental study is to evaluate the competition between the adhesion of bacteria and osteoblasts on the surface of dental implants with different morphological characteristics. By analysing cellular behaviour in the presence of pathogenic agents, we aim to highlight the crucial influence of the microtopography of the implant surface on the balance between tissue integration and bacterial colonisation. The study also emphasises the importance of strictly following therapeutic protocols during the clinical stages—from handling to insertion—to prevent bacterial contamination of the implant. In addition, the essential role of maintaining optimal oral hygiene during the post-implantation period is highlighted. Inadequate plaque control may favour biofilm formation and the occurrence of peri-implant infections, even in the case of initially favourable osseointegration. Therefore, the prevention of bacterial contamination requires both rigorous clinical measures and the active involvement of the patient in the daily care of the implanted area.

To illustrate these aspects, titanium discs with three distinct surface types were used to analyse the influence of topography on the adhesion of bacteria and osteoblasts. In this context, the behaviour of osteoblasts was investigated using pre-differentiated cells from the dental follicle, in the presence of bacterial strains—*S. oralis* ATCC 9811 and *E. faecalis* ATCC 29212. The study included both the individual evaluation of the cellular and bacterial response on the three types of surfaces and the analysis of the competitive interaction between cells and bacteria through the development of three experimental co-culture models. Separately, bacterial biofilm formation on these surfaces was quantitatively analysed in the absence of osteoblastic cells to support the findings from the bacterial cultivation experiments.

### Rationale for the Selection of Bacterial Strains

In this study, *S. oralis* and *E. faecalis* were selected due to their involvement in the etiopathogenesis of peri-implantitis. *S. oralis* is an early colonising species, playing an important role in the initiation of biofilm formation on implant surfaces, while *E. faecalis* has been frequently identified in peri-implant sites, being characterised by increased resistance to antimicrobial treatments. Both species are facultative anaerobic bacteria, which allowed their cultivation under standard laboratory conditions. The selection of these strains therefore enables a relevant and feasible investigation of the bacteria–cell interaction in the proposed model.

## 2. Materials and Methods

### 2.1. Materials

#### 2.1.1. Discs

In this study, titanium grade 4 discs (TiCP4 = Titanium Commercial Purity Grade 4) with a diameter of 6 mm and a thickness of 2 mm were used, supplied by Dentix Millennium (manufacturer: Dynamet; Washington, PA, USA).

The three types of surfaces used were machined (Ma), sandblasted, large-grit, and acid-etched (SLA), and nanostructured (Nano).

The surface obtained by precision turning (Ma) is exactly as it comes from the lathe, after sectioning from the bar stock, with a roughness of 0.8–1 μm. The lathe used was a Citizen Cincom L20m (Cincom, Esslingen, Germany). After machining, the specimens were cleaned by ultrasonication for 15 min in isopropyl alcohol (IPA) at room temperature, and then cleaned in an anionic detergent solution (Liquinox—manufacturer: Alconox, Liquinox, Alconox, New York, NY, USA) at 60 °C for 15 min with ultrasonication, followed by rinsing in ultrapure water (produced in-house using the customised Dentix Millennium system) for 15 min with ultrasonication at room temperature. Finally, specimens were dried at 60–65 °C with HEPA-filtered air.

The SLA surface has a roughness of 1–2 μm and is obtained in two steps. In the first step, the specimens are sandblasted with white electrocorundum particles of 150–200 μm grain size for 30 s at a pressure of 4–6 bar. After sandblasting, the specimens are washed in an anionic detergent solution (Liquinox—manufacturer: Alconox, USA) at 60 °C for 15 min with ultrasonication and rinsed in ultrapure water (produced in-house using the customised Dentix Millennium system) for 15 min with ultrasonication at room temperature. This is followed by acid etching with a mixture of sulfuric acid (H_2_SO_4_), 96%, and hydrochloric acid (HCl), 37%, in equal volume parts at 40 °C for 3 min. Subsequently, the specimens were rinsed in ultrapure water and immersed for 24 h in a saturated potassium bicarbonate (KHCO_3_) solution. Finally, the specimens underwent an additional ultrasonic rinse in ultrapure water at 40 °C for 30 min and were then dried at 60–65 °C with HEPA-filtered air.

The Nano surface is a patented technology developed by Dentix Millennium (invention patent issued by OSIM no. RO 133094 B1). On the surface of the specimens, a layer of TiO_2_ nanotubes is obtained, with diameters of 50–60 nm and heights of 100–200 nm, oriented perpendicularly to the surface of the implant or prepared sample, with a roughness of 1.03 µm. The specimens are first machined and decontaminated (as described above), then sandblasted and again decontaminated (as described above). Next, anodization is performed on the surface treatment line—a unique piece of equipment, Dentix NANOline—which produces the layer of nanotubes described above. A sequence of washing and rinsing steps with ultrasonication at 40–60 °C for 30 min each is carried out, and, finally, the specimens are dried at 60–65 °C with HEPA-filtered air.

#### 2.1.2. Cells and Bacterial Strains

In this study, mesenchymal stem cells (MSCs) previously isolated from the dental follicle (DF) within another project published by Lucaciu et al. (2015) [7] were used and cryopreserved until the time of use, the methods of isolating, preserving and culturing are described in detail in another work [7]. Adult bone tissue consists of three main types of cells: osteoblasts and osteocytes—both derived from mesenchymal stem cells (MSCs)—and osteoclasts, which originate from hematopoietic stem cells (HSCs).

In maintaining bone homeostasis, each of these cells has a specific role: osteoblasts contribute to the formation and mineralization of bone tissue, osteoclasts are responsible for the resorption and degradation of the bone matrix, while osteocytes—mature cells embedded within the matrix—regulate the activity of the other two cell types, thus ensuring the structural and functional integrity of the bone [8,9,10]. The bacterial strains used in this study were purchased from Thermo Fisher Scientific (Waltham, MA, USA).

### 2.2. Methods

#### Structure and Chemical Composition of the Discs

The morphology of the three types of discs was evaluated using a TESCAN VEGA scanning electron microscope (Brno, Czech Republic) operated at an accelerating voltage of 15 keV. Energy-dispersive spectroscopy (EDS) analysis was performed using the same equipment and operating conditions following molybdenum sputter coating at a beam current of 61 pA.

### 2.3. Preparation of the Discs

The Ti discs were individually packed and sealed in sterilisation pouches (Dr. Mayer, Foshan, Guangdong, China) and sterilised by autoclaving, using saturated steam under pressure (135 °C, 2.15 bar, for 20 min), according to the standard moist-heat sterilisation protocol. The equipment used was the Dxp Domina autoclave, programme B134 (DentalX, Thiene, Italy).

### 2.4. Optimisation of Dental Follicle Mesenchymal Stem Cell Culture on Titanium Discs

To optimise the experimental conditions for subsequent co-culture with bacterial cells, which require distinct cultivation parameters, preliminary tests were performed under CO_2_-free incubation conditions using Leibovitz’s L-15 medium (Sigma-Aldrich, St. Louis, MO, USA). This medium is specifically formulated for CO_2_-independent systems, containing additional sodium bicarbonate, salts, free-base amino acids, and galactose—which replaces glucose to ensure physiological pH stability. L-15 medium supports a variety of established cell lines (such as HEp-2 and LLC-MK2) as well as primary explants of both embryonic and adult human tissues. The complete medium was adapted from the standard stem cell formulation, consisting of L-15 medium supplemented with 15% foetal bovine serum, 1% antibiotics, 1% L-glutamine, 1% non-essential amino acids, 55 μM β-mercaptoethanol, and 1 mM sodium pyruvate (Sigma-Aldrich, USA).

Cell viability of DF cells was assessed using the FDA assay. The culture medium was adapted from a standard stem cell medium formulation and consisted of L-15 medium supplemented with 15% foetal calf serum, 1% antibiotics, 1% L-glutamine, 1% non-essential amino acids, 55 μM β-mercaptoethanol, and 1 mM sodium pyruvate (Sigma-Aldrich, USA). Cells were incubated at 37 °C in a CO_2_-free atmosphere. To determine cell viability in real time, 72 and 96 h after seeding, cells were vitally stained with fluorescein diacetate (FDA) (Sigma-Aldrich, USA). MSCs derived from dental follicles that adhered to the titanium disc surfaces were incubated for 5 min at 37 °C in the dark with 800 µL/well of FDA solution, prepared at a final concentration of 2.4 µM in PBS supplemented with Ca^2+^ and Mg^2+^ ions. After incubation, the wells were washed twice with PBS and the implants were analysed on a Zeiss Axio Observer D1 inverted fluorescence microscope using a 488 nm filter. Images were captured with a CCD camera (Axiocam MRM) adapted to the microscope and analysed using version 4.6.3 of Axiovision software.

### 2.5. Co-Culture Experiments of Osteogenically Pre-Differentiated Dental Follicle Mesenchymal Stem Cells and Bacteria

For the co-culture experiments, mesenchymal stem cells were pre-differentiated for two passages in osteogenic medium.

The co-culture was performed in 48-well plates in which the three types of titanium discs were placed. Osteogenically pre-differentiated cells were trypsinized and centrifuged. Cell counting was performed using an automatic cell counter (EVE™). Dental follicle-derived (DF) cells were seeded at a density of 1 × 10^5^ cells per disc in 350 µL of medium per well. The culture medium consisted of an osteogenic formulation without antibiotics adapted to the new conditions: L-15 medium supplemented with 15% foetal bovine serum, 1% L-glutamine, 1% non-essential amino acids, 10 µM dexamethasone, 10 mM β-glycerophosphate, and 50 µg/mL ascorbic acid (Sigma-Aldrich, USA).

The experimental design was established as follows:

- Implants with DF pre-differentiated cells only (1 × 10^5^ cells per implant);
- Implants with bacterial cells only (from a bacteria cell suspension of 2 × 10^8^ PKH26 stained bacteria/mL 10 μL was added per implant reaching to 2 × 10^6^ bacterial cells/implant. The ratio between DF pre-differentiated cells/bacteria was 1/20);
- Co-culture with DF pre-differentiated cells and bacteria seeded simultaneously at the same cell concentrations (Model A);
- Co-culture with dental DF pre-differentiated cells followed by bacterial seeding after two hours at the same cell concentrations (Model B);
- Co-culture with bacteria seeded first and DF pre-differentiated cells added after two hours at the same cell concentrations (Model C).

Bacterial cells (*S. oralis* ATCC 9811 and *E. faecalis* ATCC 29212) were previously vital-stained using the PKH26 Red Fluorescent Cell Linker Kit (Sigma-Aldrich). PKH26 is a lipophilic fluorescent dye commonly used for membrane staining, emitting red fluorescence (Ex/Em = 551/567 nm). This dye is primarily employed for in vitro cell labelling and intercalates into the lipid bilayer of cell membranes, providing stable and long-lasting fluorescence.

Initially, bacteria were cultured on Columbia blood agar plates containing 5% sheep blood (BioMérieux, Marcy-l’Étoile, France) for 24 h at 37 °C. Using freshly grown colonies, a bacterial suspension of 0.6 McFarland was prepared in physiological saline (0.6 McF corresponds to approximately 1.8 × 10^8^ CFU/mL). After enumeration, bacterial cells were centrifuged at 3000 rpm for 15 min. The bacterial pellet was resuspended in 2 mL diluent to which 2 × 10 µL of PKH26 dye was added. The bacteria were incubated for 5 min at room temperature, after which 10 mL of Leibovitz’s (L-15) medium containing 10% foetal serum was added to each tube, and samples were centrifuged again for 15 min at 3000 rpm. A further wash with complete medium was performed, and the cells were resuspended in 2 mL of medium and seeded onto the surface of the titanium discs.

DF cells and bacterial cells were then seeded on the titanium discs using L-15 medium without antibiotics and incubated at 37 °C in CO_2_-free incubators. The incubation time for both co-cultures and individual cell/bacterial cultures on titanium discs was 2, 24, and 48 h. At the end of each incubation period, the cells were fixed with 4% paraformaldehyde after three washes with PBS. Following fixation, three additional PBS washes were performed, and DAPI (4′,6-diamidino-2-phenylindole) (Sigma-Aldrich) was added at a concentration of 1 µg/mL to stain the nuclei of dental follicle-derived pre-differentiated cells.

The fixed implants were examined under a Nikon Eclipse 600 fluorescence microscope (Nikon, Bangkok, Thailand) using a 375/28 nm filter for DAPI and a 540/25 nm filter for PKH26-stained bacteria. Images were captured with a digital camera and analysed using NIS Elements Nikon Image Software (Kensington, MD, United States), using the same image acquisition parameters. Three selected fields in the same pre-designated implant areas were analysed. Nuclei of DF-derived cells and bacterial cells were quantified using ImageJ (v1.52P, NIH), all tests used biological triplicate.

To simulate clinically relevant scenarios of implant site contamination, three experimental co-culture models were developed, defined by different timings of bacterial exposure relative to cellular colonisation.

Model A—simultaneous exposure (intraoperative contamination or contaminated post-extraction socket).

Cells and bacteria were seeded simultaneously on titanium discs to reproduce both intraoperative contamination of the implant site and situations where implants are immediately inserted post-extraction into a potentially bacteria-colonised socket.

Objective: to evaluate the initial competition for surface colonisation (“race for the surface” concept) and the immediate effect of bacteria on cell adhesion and seeding.

Model B—cells first, bacteria after two hours (delayed bacterial colonisation).

Cells were seeded first, and bacteria were added after two hours to reproduce bacterial colonisation occurring after implant insertion, in the context of reduced local immunity or poor oral hygiene.

Objective: to assess the stability of initial cell adhesion and its ability to limit subsequent bacterial attachment.

Model C—bacteria first, cells after two hours (implant pre-contaminated before insertion).

Titanium surfaces were first exposed to bacteria; after two hours, cells were added. This model experimentally reproduces situations in which the implant is accidentally contaminated during surgical procedures.

Objective: to investigate the effect of early biofilm formation on subsequent cell colonisation.

### 2.6. Evaluation of Biofilm Formation

The biofilm developed on the surface of the titanium discs was analysed using a modified and adapted crystal violet staining technique. This method is based on the ability of the dye to bind to the extracellular components of the biofilm, particularly extracellular polysaccharides and proteins. Thus, the presence of the biofilm can be easily visualised, and its quantity can be estimated semi-quantitatively. The main advantage of this technique lies in its simplicity, allowing simultaneous analysis of multiple samples at relatively low cost.

In this experiment, the amount of biofilm formed by *S. oralis* ATCC 9811 and *E. faecalis* ATCC 29212 was determined. Initially, bacteria were cultured on Columbia agar medium containing 5% sheep blood (BioMérieux, Marcy-l’Étoile, France) for 24 h at 37 °C. Using freshly grown bacterial colonies, a suspension corresponding to a density of 0.5 McFarland was prepared in physiological saline (0.5 McF corresponds to approximately 1.5 × 10^8^ CFU/mL).

For biofilm formation, 2 mL of bacterial suspension and 2 mL of Mueller Hinton broth (MBH, Bio-Rad, Marnes-la-Coquette, France) were added over the titanium discs. Samples were incubated at 37 °C, and the total amount of bacterial biofilm developed was evaluated after 96 h.

Following incubation, the discs with biofilm were washed three times with sterile physiological saline to remove planktonic cells and residual debris. The biofilm formed on the dental implant discs was stained with 1% crystal violet solution for 2 min. To remove excess dye, the samples were washed again three times with sterile physiological saline. After drying, the biofilm was solubilised using ethyl alcohol. The resulting solution was transferred into glass cuvettes, and absorbance was measured using a UV–VIS spectrophotometer at 590 nm. The assay used biological triplicates.

### 2.7. Statistical Analysis

Cell and bacterial counts were performed using the ImageJ software. The obtained results were processed in Excel to calculate the mean value, standard deviation, and to generate graphs. Statistical analysis was carried out using one-way and two-way analysis of variance (ANOVA) with SPSS (Version 11.5, SPSS, Chicago, IL, USA) software package, with a Bonferroni test with the level of significance set at 0.05 to calculate the significant differences between the mean values.

## 3. Results

### 3.1. Structure and Chemical Characterisation of the Discs

**Figure 1:**
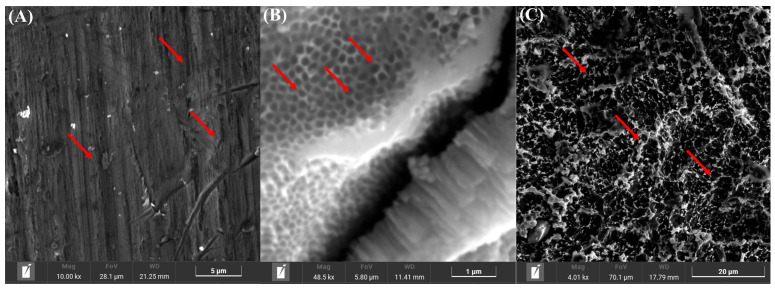
SEM imagines of the discs (red arrows point the typical characteristics of each surface): (**A**) Ma; (**B**) Nano; (**C**) SLA.

The SEM analysis of the discs revealed for the Ma surfaces a typical lathe cut (Figure 1A) with a relatively smooth surface, pointed by the red arrows. The investigation for the Nano surface (Figure 1B) shows tubes perpendicularly oriented on the surface of the samples. For what concerns the SLA disc the SEM imagines highlight a surface covered by irregularities (Figure 1C), obtained after the sandblasting and acid etching process.

**Figure 2:**
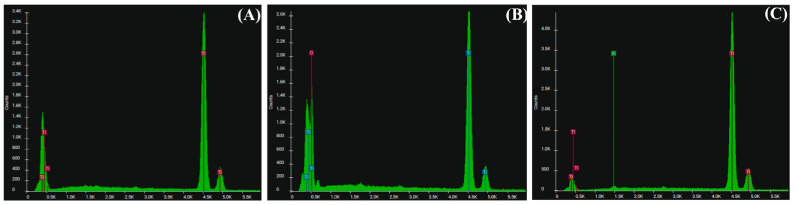
EDS analysis: (**A**) Ma; (**B**) Nano; (**C**) SLA.

The diffractogram from the EDS investigation showed diffraction peaks for the discs Ma and SLA typical for titanium (Figure 2A,C), with a very low content of aluminium (0.01% in weight) for SLA. Regarding the Nano surface, peaks standing for oxygen and titanium (Figure 2B) were evidenced.

### 3.2. Evaluation of the Adhesion of Pre-Differentiated DF Cells on the Three Types of Titanium Surfaces

**Figure 3:**
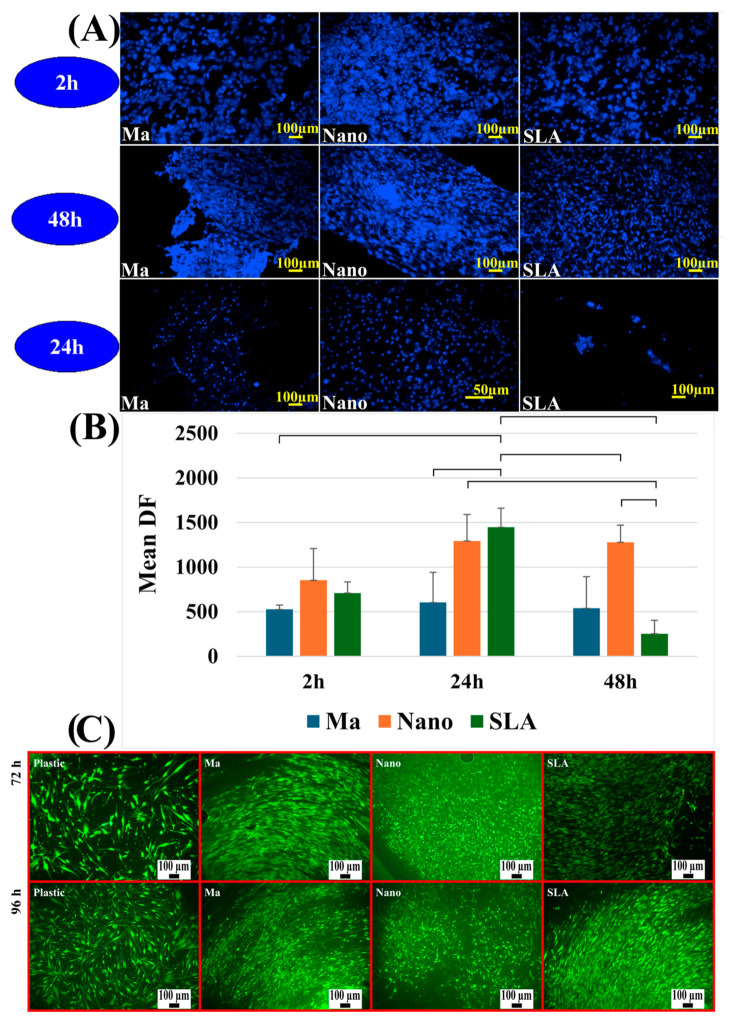
(**A**) Fluorescence microscopy images of pre-differentiated DF cells on the three surfaces Ma, Nano, and SLA at 2 h, 24 h, and 48 h; (**B**) mean of cells recorded on the three surfaces at different time intervals (with statistically significant differences using Bonnferoni test); (**C**) morphological appearance in fluorescence microscopy of DF cells cultured in L-15 medium adapted in CO_2_ free conditions. Staining with fluorescein diacetate. Magnification (objective ×10).

Fluorescence microscopy images revealed differences in cell density according to surface type (Figure 3A,B). It can be clearly seen that the SLA surface experiences an expansion in cell population at 24 h, followed by a dramatic decrease at 48 h. The Ma surface shows a very slow, constant cell grow, in contrast to the Nano surface trend, where a steady growth over the hours is evident, maintaining the highest overall proliferation level at the final 48 h time point. The two-way Anova shows that the surface, time and their interaction effect have a statistically significant impact on the cell count with F = 5.883, *p* = 0.003 (Figure 3B).

The viability of cells grown on titanium surfaces and compared to the standard plastic surface was evaluated by vital staining with fluorescein diacetate for cells grown in adapted L-15 medium for 72 and 96 h under CO_2_ free conditions. Compared to the plastic control, cell viability was preserved for all cells grown on titanium surfaces, with increased cell proliferation especially for SLA and Nano discs. Differences in cell morphology were also observed. Thus, for Ma and SLA discs, a fibroblastoid-like shape was visualised, in contrast to cells grown on the Nano surface which adopted a more polygonal, flattened shape (Figure 3C).

### 3.3. Evaluation of the Adhesion of E. faecalis and S. oralis on the Three Types of Titanium Surfaces

**Figure 4:**
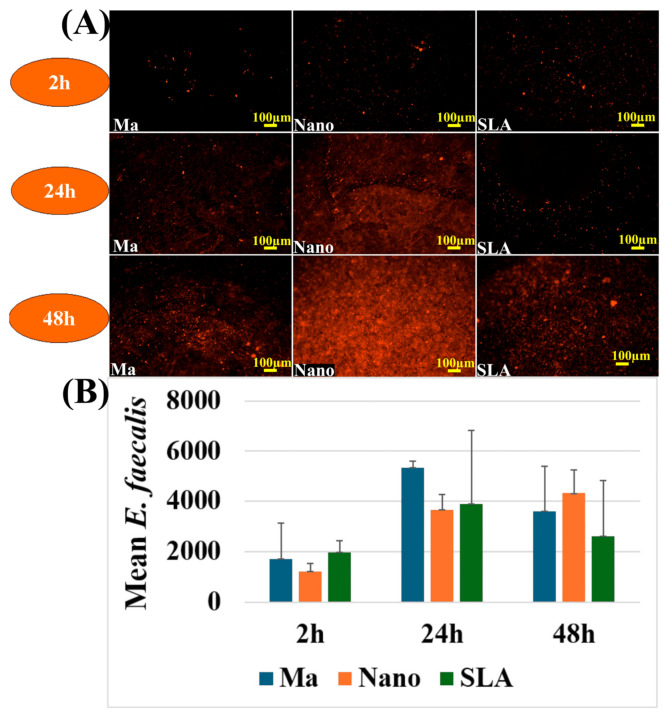
(**A**) Fluorescence microscopy images of *E. faecalis* on the three surfaces Ma, Nano, and SLA at different time intervals; (**B**) mean of *E. faecalis* recorded on the three surfaces at different time intervals.

Fluorescence microscopy images confirmed the bacterial quantification results for E. faecalis (Figure 4A,B), revealing clear differences among the surface types. At 2 h, bacterial density was low on all surfaces, with discrete and relatively uniform adhesion. At 24 h, colonisation was much more pronounced on the Ma surface, where evident bacterial clusters were observed, while the Nano surface displayed reduced bacterial presence, and the SLA surface showed an intermediate level. At 48 h, colonisation intensified on all surfaces, with extensive coverage and the appearance of dense bacterial clusters suggestive of microcolony formation and early biofilm development; however, morphological differences among the Nano, Ma, and SLA surfaces were less evident (Figure 4B). The type of material used does not show a statistically verifiable impact on the results, nor does it alter how the values change over time (F = 0.86; *p* = 0.05), but there is a statistically significant change in value over time, driven by a significant increase from 2 h to 24 h, which remains elevated at 48 h (F = 7.45; *p* = 0.004).

**Figure 5:**
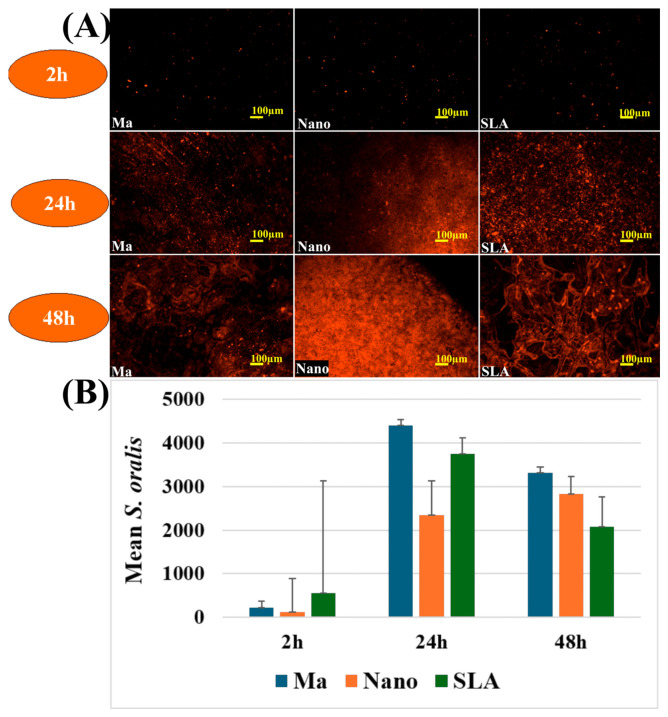
(**A**) Fluorescence microscopy images of *S. oralis* on the three surfaces Ma, Nano, and SLA at different time intervals; (**B**) mean of *S. oralis* of recorded on the three surfaces at different time intervals.

For *S. oralis* the fluorescence microscopy images showed a progressive increase in bacterial density on all surfaces, with a relatively low and uniform distribution at 2 h. At 24 h, larger bacterial clusters became evident, particularly on the Ma and SLA surfaces, while the Nano surface still displayed lower bacterial presence. At 48 h, colonisation became abundant on all surfaces, with the appearance of dense bacterial aggregates suggestive of microcolony formation and consistent with the early organisation of a biofilm (Figure 5A,B). Similar to *E. faecalis*, the statistical analysis demonstrated that time was the only factor significantly influencing the recorded values (F = 7.457, *p* = 0.004). This finding was supported by the marked increase observed from 2 h to 24 h and 48 h across all three types of discs.

### 3.4. Evaluation of Model A—Co-Culture with Pre-Differentiated DF Cells and Bacteria Seeded Simultaneously

The results obtained from the simultaneous seeding of DF cells and *E. faecalis* bacteria showed clear differences among the tested surface types.

**Figure 6:**
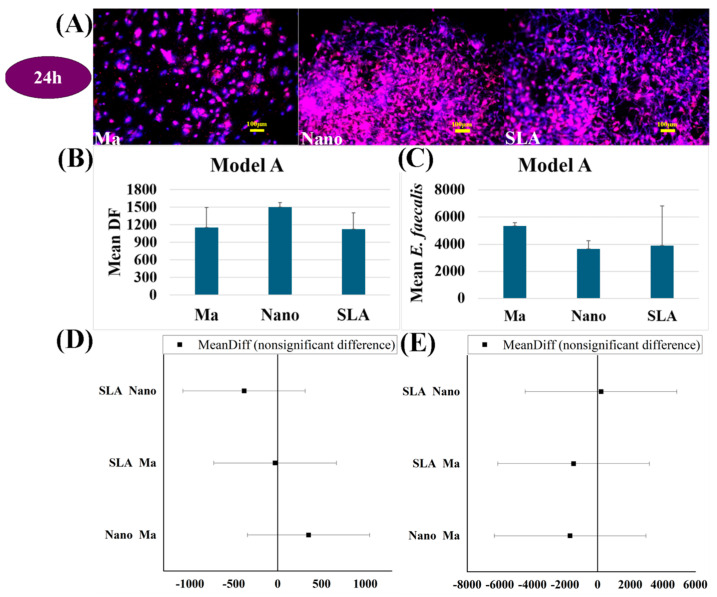
(**A**) Fluorescence microscopy images of *E. faecalis* and pre-differentiated DF cells on the three surfaces Ma, Nano, and SLA at 24 h; (**B**) mean of pre-differentiated DF cells recorded on the three surfaces during the simultaneous seeding *E. faecalis* and pre-differentiated DF cells; (**C**) mean of *E. faecalis* recorded on the three surfaces at different during the simultaneous seeding of *E. faecalis* and pre-differentiated DF cells; (**D**) mean difference in *E. faecalis* (ANOVA one way (Bonferroni = 0.05)); (**E**) mean difference in DF (ANOVA one way (Bonferroni = 0.05)).

Regarding cell adhesion, the Nano surface exhibited the highest number of adherent cells at 24 h (Figure 6A,B), but with no statistically significant differences compared with the other two (Figure 6B).

In the case of the *E. faecalis* on the contrary, the number of bacteria was lower than all other surfaces, without a statistically significant difference regarding the other surfaces (Figure 6C).

**Figure 7:**
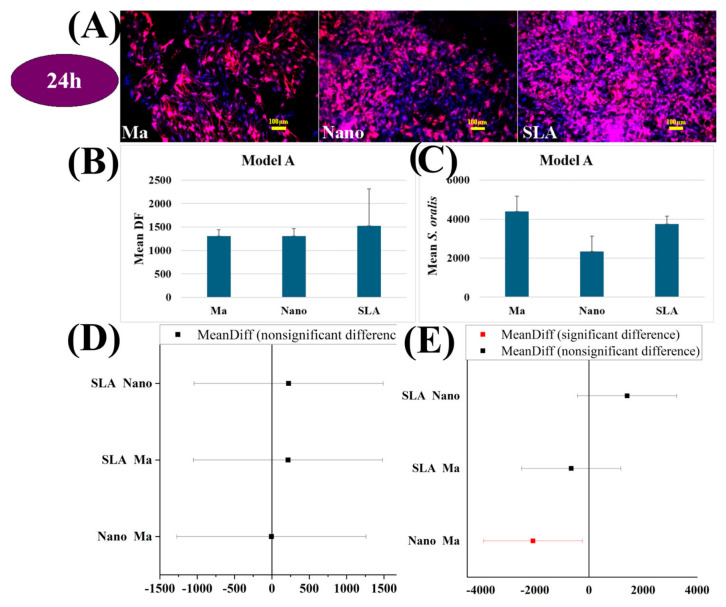
(**A**) Fluorescence microscopy images of *S. oralis* and pre-differentiated DF cells on the three surfaces Ma, Nano, and SLA at 24 h; (**B**) mean of DF recorded on the three surfaces during the simultaneous seeding of *S. oralis*; (**C**) mean of *S. oralis* recorded on the three surfaces; (**D**) mean difference in *E. faecalis* (ANOVA one way (Bonferroni = 0.05)); (**E**) mean difference in DF (ANOVA one way (Bonferroni = 0.05)) (co-culture Model A).

Fluorescence microscopy images of the co-culture between pre-differentiated DF cells and *S. oralis* (Model A) showed a differentiated dynamic among the surfaces (Figure 7). At 24 h, both cells and bacteria became more numerous on all surfaces; however, on Ma and SLA a more pronounced bacterial presence was evident. A statistical difference being observed between the Nano and Ma disc (Figure 7E). For the DF cells, the results show a slightly higher proliferation on the SLA surface, but without statistical difference regarding the other two samples (Figure 7B).

### 3.5. Evaluation of Model B—Cells First, Bacteria After Two Hours (Delayed Bacterial Colonisation)

**Figure 8:**
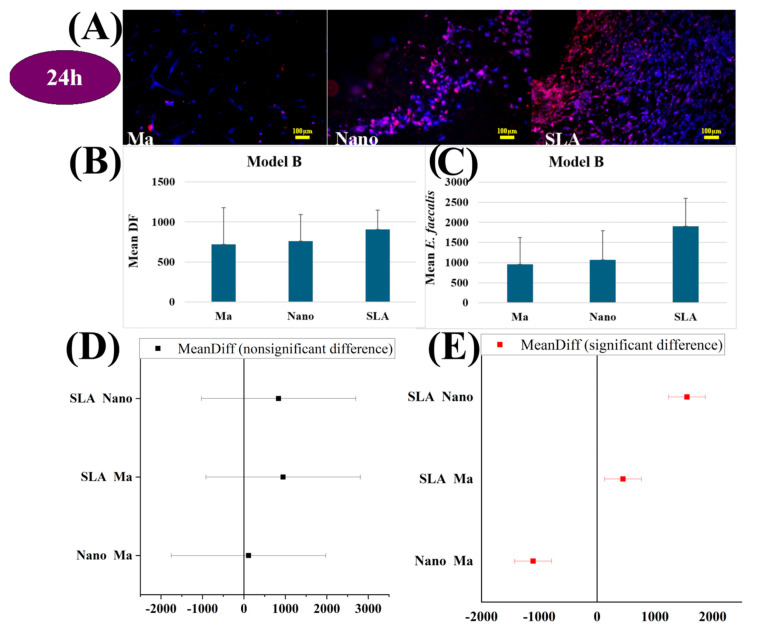
(**A**) Fluorescence microscopy images of *E. faecalis* and pre-differentiated DF cells on the three surfaces Ma, Nano, and SLA at 24 h (co-culture Model B); (**B**) mean of pre-differentiated DF cells recorded on the three surfaces in the case of Model B co-culture with *E. faecalis*, at 24 h; (**C**) number of *E. faecalis* recorded on the three surfaces in the case of Model B co-culture, at 24 h; (**D**) mean difference in DF (ANOVA one way (Bonferroni = 0.05)); (**E**) mean difference in *E. faecalis* (ANOVA one way (Bonferroni = 0.05)).

Fluorescence microscopy images showed good cellular coverage on all surfaces, while bacterial colonisation was moderate on Nano and highest on SLA (Figure 8A) for the co-culture involving *E. faecalis*. For the pre-differentiated DF cells, no statistically significant differences were recorded between the surfaces; however, the SLA surface showed a slightly increased tendency for adhesion (Figure 8B,E). Under the conditions of Model B co-culture, at 24 h, bacterial colonisation was more intense on the SLA surface, while on Nano and Ma surfaces, bacterial levels were lower, with statistically significant difference for *E. faecalis* on all three surfaces (Figure 8C,D).

**Figure 9:**
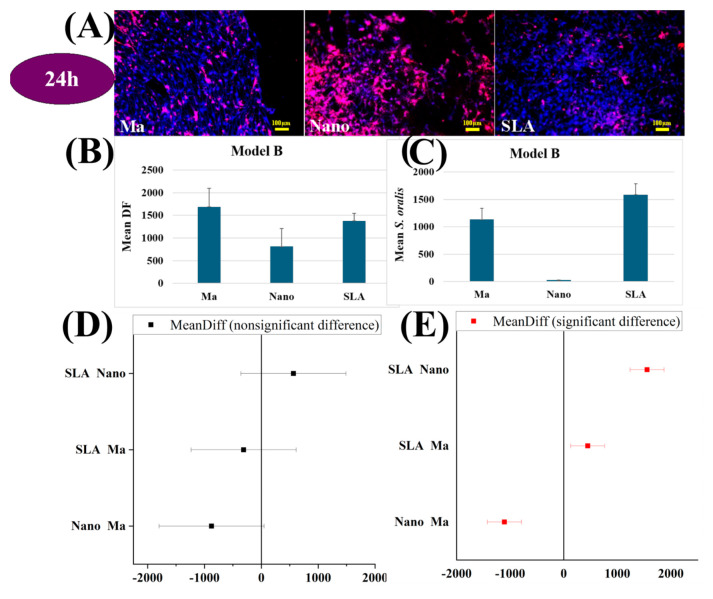
(**A**) Fluorescence microscopy images of *S. oralis* and pre-differentiated DF cells on the three surfaces Ma, Nano, and SLA at 24 h (co-culture Model B); (**B**) mean of pre-differentiated DF cells recorded on the three surfaces in the case of Model B co-culture with *S. oralis*, at 24 h; (**C**) number of *S. oralis* recorded on the three surfaces in the case of Model B co-culture, at 24 h; (**D**) mean difference in *S. oralis* (ANOVA one way (Bonferroni = 0.05)); (**E**) mean difference in DF (ANOVA one way (Bonferroni = 0.05)).

Fluorescence microscopy images revealed significant differences in the distribution of cells and bacteria on the three types of analysed surfaces: Ma, Nano, and SLA in the case of co-culture with *S. oralis* (Figure 9). The Ma surfaces exhibited extensive cellular coverage associated with a moderate bacterial presence. The Nano surfaces were characterised by a reduced density of both cells and bacteria, whereas the SLA surfaces showed a more uniform distribution with a visible presence of both types of structures (Figure 9A). The number of DF cells present on the three different surface types showed no statistically significant differences (Figure 9D), in contrast to the number of bacteria detected on the Ma, Nano, and SLA discs (Figure 9E).

### 3.6. Evaluation of Model C—Bacteria First, Cells After Two Hours (Implant Pre-Contaminated Before Insertion)

**Figure 10:**
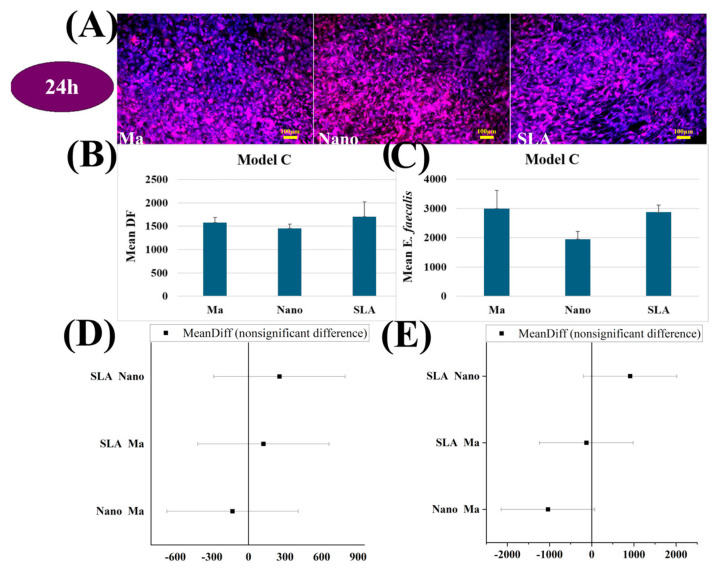
(**A**) Fluorescence microscopy images of *E. faecalis* and pre-differentiated DF cells on the three surfaces Ma, Nano, and SLA at 24 h (co-culture Model C); (**B**) mean of pre-differentiated DF cells recorded on the three surfaces in the case of Model C co-culture with *E. faecalis*, at 24 h; (**C**) number of *E. faecalis* recorded on the three surfaces in the case of Model C co-culture, at 24 h; (**D**) mean difference in DF (ANOVA one way (Bonferroni = 0.05)); (**E**) mean difference in *E. faecalis* (ANOVA one way (Bonferroni = 0.05)).

Microscopy images for Model C co-culture revealed bacterial colonisation, regarding *E. faecalis on* all surfaces, with a comparable appearance on Ma and SLA (Figure 10A). Regarding the cells, the observed distribution was consistent with the graphical results, remaining relatively uniform across the surfaces. The pre-differentiated DF cells adhered to all three surfaces, with slightly higher values on SLA and Ma, but without statistically significant differences compared with Nano (Figure 10D). However, SLA showed a slightly increased tendency for adhesion compared with the other surfaces (Figure 10B). In the case of Model C co-culture, the number of *E. faecalis* bacteria was lowest on the Nano surface. On MA and SLA, bacterial levels were comparable and slightly higher than on Nano (Figure 10C).

**Figure 11:**
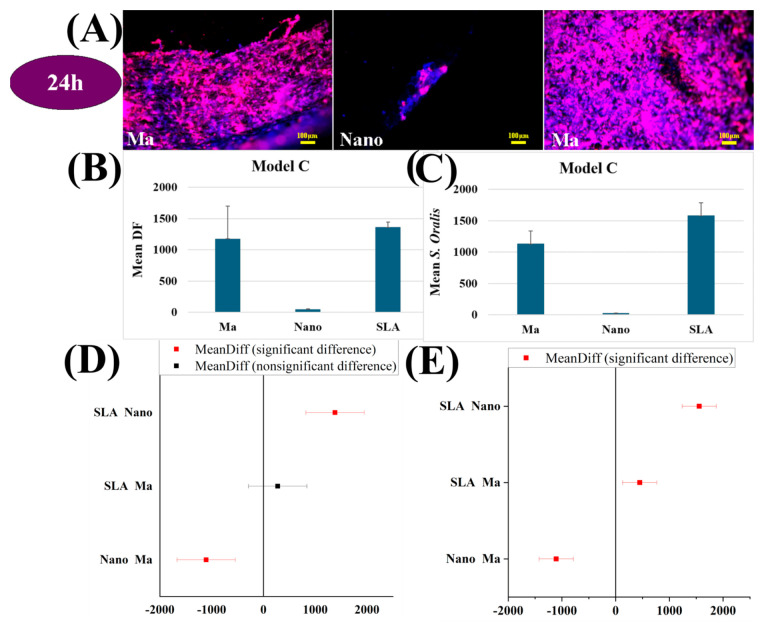
(**A**) Fluorescence microscopy images of *S. oralis* and pre-differentiated DF cells on the three surfaces Ma, Nano, and SLA at 24 h (co-culture Model C); (**B**) mean of pre-differentiated DF cells recorded on the three surfaces in the case of Model C co-culture with *S. oralis,* at 24 h; (**C**) mean of *S. oralis* recorded on the three surfaces in the case of Model C co-culture with *S. oralis,* at 24 h; (**D**) mean difference in DF (ANOVA one way (Bonferroni = 0.05)); (**E**) mean difference in *S. oralis* (ANOVA one way (Bonferroni = 0.05)).

Fluorescence microscopy images revealed significant differences in the distribution of cells and bacteria on the three types of analysed surfaces: Ma, Nano, and SLA for *S. oralis*. The Ma surfaces exhibited extensive cellular coverage associated with a moderate bacterial presence. The Nano surfaces were characterised by a reduced density of both cells and bacteria, whereas the SLA surfaces showed a more uniform distribution with a visible presence of both types of structures (Figure 11A). In the case of the Model C co-culture with *S. oralis*, bacterial adhesion was significantly higher on the SLA surface, followed by Ma. The Nano surface showed the lowest values, highlighting that *S. oralis* adheres least efficiently to this type of surface (Figure 11C). Like the results obtained for bacteria, the Nano surface exhibited the lowest levels of cellular adhesion, with the difference compared with the other surfaces being statistically significant. On the Ma and SLA surfaces, cell density was higher and relatively similar (Figure 11B).

### 3.7. Evaluation of the Biofilm Formed on the Three Surfaces

**Figure 12:**
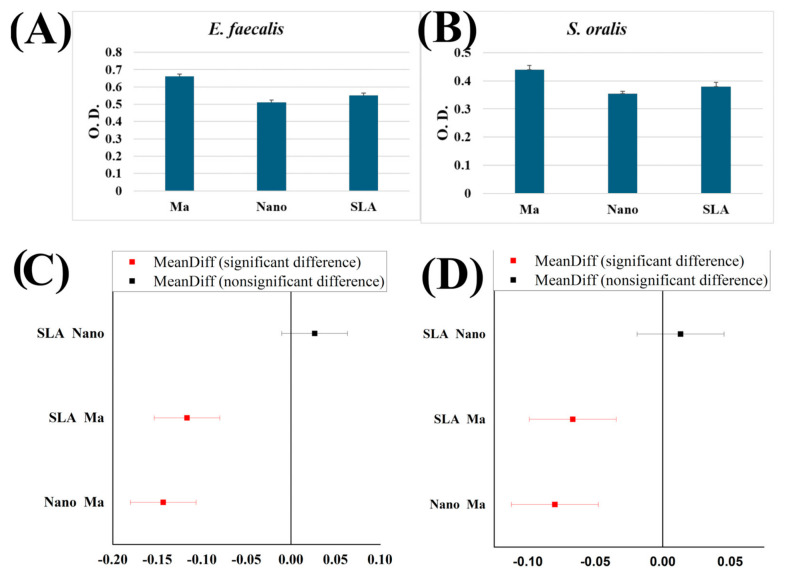
Biofilm formation of (**A**) *E. Faecalis* and (**B**) *S. Oralis;* (**C**) mean difference in *E. faecalis* (ANOVA one way (Bonferroni = 0.05)) and (**D**) mean difference in *S. oralis* (ANOVA one way (Bonferroni = 0.05)) on Ma, Nano, SLA at 96 h.

On all three types of tested surfaces (Ma, SLA, and Nano), both bacterial species were able to develop biofilm. However, the degree of biofilm formation differed between *E. faecalis* and *S. oralis*, suggesting distinct mechanisms of adaptation and colonisation. *E. faecalis* produced a greater amount of bacterial biofilm at 96 h on all three types of dental implants: Ma (O.D. = 0.66), SLA (O.D. = 0.55), and Nano (O.D. = 0.51). The smallest amount of biofilm produced by *E. faecalis* was observed on the Nano implant (Figure 12). Regarding *S. oralis*, the trend was similar, with the highest amount of biofilm recorded on the Ma implant (O.D. = 0.44) and the lowest on the Nano implant (O.D. = 0.36) (Figure 12). The statistical analysis showed for *E. faecalis* values with a statistical difference between the discs SLA and Ma, respective Nano and Ma (Figure 12C), the trend being the same also for *S. oralis* (Figure 12D).

## 4. Discussion

### 4.1. Main Findings—Consistencies and Discrepancies with Previous Results

The interaction between dental implant surfaces and the biological environment is governed by a series of physicochemical and structural factors that influence both bacterial adhesion and cellular behaviour. Surface roughness, and micro- and nano-scale topography of dental implants can either promote or, conversely, limit bacterial colonisation, while simultaneously affecting the osseointegration process by modulating how osteoblasts adhere, proliferate, and differentiate. Therefore, the characteristics of the implant surface may represent both a favourable factor for osseointegration and a potential risk for bacterial colonisation and the development of peri-implant infections [1,11,12,13]. The structural investigation of the tested materials showed high differences in surface characteristics, a fact reflected also in the Ra values of the discs (Ma = 0.8–1 µm; Nano = 1.03 µm; SLA = 1–2 µm), the Ma disc being relatively smooth, when compared with SLA surface, and the Nano one being covered by tubes (Figure 1). The EDS analyses (Figure 2) showed only titanium at the surface of the Ma, in contrast to the Nano, which revealed titanium and oxygen. Regarding the SLA the quantity analysis show a drop of aluminium (0.01% in weight) beside titanium.

To better understand these interactions, we developed experimental osteoblast–bacteria co-culture models, similar to those reported in other published studies [2,5,6,14]. These models simulate different clinical scenarios: simultaneous colonisation of the surface by bacteria and cells (Model A), as well as alternative situations in which one population gains an initial colonisation advantage (Model B and Model C). Through this approach, it is possible to assess the competitive differences between osteoblasts and bacteria depending on the surface type, providing relevant insights for selecting the most appropriate surface treatments to reduce infection risk and promote cell adhesion. Given that peri-implant infections represent one of the main causes of implant failure, these findings reinforce the idea that choosing the optimal surface must always be accompanied by strict control of the surgical environment [3].

Two bacterial species with distinct yet complementary roles in implant surface colonisation were selected. *E. faecalis* is an opportunistic bacterium frequently associated with persistent oral infections and known for its ability to form resistant biofilm, making it a suitable model for assessing the risk of chronic implant contamination [15,16]. In addition, this species can survive post-extraction, in a vegetative state, within healed bone, from which it may be reactivated at the time of implant placement, subsequently contributing to implant colonisation [17,18]. Our results showed that *E. faecalis* initially adhered to all three types of tested surfaces; however, its subsequent behaviour differed significantly. On the Nano surface, the number of initial adherent bacteria was low, the proliferation at 24 was high for all three surfaces, but at 48 h the proliferation rate decreased, suggesting a reduced capacity for proliferation and survival. These observations being similar to other studies [17,19]. Moreover, *E. faecalis* biofilm has been reported to induce corrosion of titanium surfaces, emphasising the direct impact of bacteria–material interactions on implant longevity [20]. The limited colonisation observed on the Nano surface at 2 h, and 24 h can be attributed to its topographical characteristics. Studies have shown that nanometric structures can significantly reduce bacterial adhesion and interfere with biofilm stability by altering the contact interface [21,22].

With regard to *S. oralis*, our results demonstrated that it exhibited low initial adhesion on the Nano surface during the first 24 h; however, this trend did not persist over time, as bacterial density slightly increased at 48 h. In contrast, bacterial levels remained highest on the Ma surface, suggesting greater tolerance of this topography for streptococcal colonisation. This behaviour is consistent with the literature, which describes *S. oralis* as an early coloniser within the oral and peri-implant biofilm [23]. As a primary colonising species, *S. oralis* facilitates the subsequent adhesion of other bacteria—including pathogens associated with peri-implantitis—through the production of extracellular matrix components and the creation of a favourable environment for later bacterial colonisation [24,25]. Conversely, the persistence of higher levels on Ma and SLA surfaces may represent an increased risk for the initiation of complex biofilm formation—an aspect supported by other clinical and experimental studies [26,27,28,29].

The results obtained from the 24 h co-culture models highlighted the essential role of colonisation order in the competition between host cells and bacteria on implant surfaces, confirming previously described concepts regarding the “race for the surface” [2,4,5,6].

In co-culture Model A, simulating intraoperative contamination through simultaneous exposure of cells and bacteria, the Nano surface proved more favourable to FD derived cells while limiting bacterial colonisation—an observation consistent with Piñera-Avellaneda et al. (2021) [2], who demonstrated that osteoblasts predominate over bacteria on bioactive surfaces, and Camargo et al. (2020) [11], who reported that functional surface treatments reduce bacterial adhesion and stimulate osteoblastic activity. On the Ma surface, bacterial levels were the highest for both species, while pre-differentiated DF cells maintained densities similar to Nano. This behaviour reflects the findings of Robles et al. (2023) [1]. The SLA surface exhibited an intermediate behaviour, without a clear dominance between cells and bacteria, suggesting that colonisation balance is also influenced by material properties beyond surface roughness. This observation aligns with Zhao et al. (2014) [5], who demonstrated that the chemical properties of the material can have a stronger influence on osteoblast survival than surface roughness.

Model B (delayed bacterial exposure, after 2 h) emphasised the importance of the temporal advantage of cells in competing with bacteria. When pre-differentiated DF cells colonised the implant surface first, they appeared to significantly reduce the likelihood of subsequent bacterial adhesion. This effect was most evident on the Nano surface, where the structure supported the formation and maintenance of a stable cell layer, while bacterial proliferation remained limited—even for aggressive species such as *E. faecalis*—consistent with findings from Gil & Sanz (2025) [30]. On SLA surfaces, although DF cells initially adhered successfully, subsequent bacterial colonisation was more pronounced, confirming the literature data [Robles et al., 2023] [1]. On the Ma surface, both bacterial and cellular levels remained intermediate, with no major differences favouring either population. This model demonstrates that the temporal advantage of cells considerably limits bacterial colonisation capacity, and that this protective effect is enhanced by Nano topography, which supports cell layer stability and discourages subsequent bacterial adhesion [Robles et al., 2015; Gil & Sanz, 2025] [1,30].

Model C, where bacteria were seeded first, reflects clinical scenarios in which the implant is placed in a contaminated socket or a site with a pre-existing microbial load. At 24 h, bacteria benefitted from a clear advantage: the SLA surface supported more persistent colonisation, particularly for *S. oralis*, while bacterial levels on Nano remained the lowest, even though pre-differentiated DF cells struggled to compete once the biofilm had consolidated. On SLA and Ma surfaces, bacterial and cellular levels were relatively similar, suggesting that under initial bacterial contamination neither topography offers a net advantage to DF cells. These results show that, although Nano reduces bacterial proliferation, when bacteria colonise first, cells have difficulty regaining dominance—an observation consistent with the literature showing that the presence of an early biofilm compromises subsequent cell adhesion [Arciola et al., 2018; Zhao et al., 2014] [3,5].

Comparative analysis of the three co-culture models underscores the crucial role of colonisation order in the competition between host cells and bacteria on implant surfaces. When cells and bacteria were exposed simultaneously (Model A), the Nano surface clearly favoured pre-differentiated DF cells while reducing bacterial colonisation. This behaviour aligns with Piñera-Avellaneda et al. (2021) [2] and Camargo et al. (2020) [11], who reported that bioactive surfaces support cellular activity and inhibit bacterial adhesion. When cells initiated colonisation first (Model B), their advantage was consolidated, and the protective effect was most evident on Nano, where the nanometric structure supported the formation of a stable cell layer and limited subsequent microbial adhesion—consistent with Gil & Sanz (2025) [30], who demonstrated that Nano surfaces enhance osseointegration. Conversely, when bacteria colonised first (Model C), they gained a clear competitive advantage, especially on SLA, where bacterial levels remained high, consistent with reports that rougher surfaces promote bacterial colonisation, as described by Robles et al. (2023) [1]. Ma surfaces exhibited an intermediate profile in all models, without a clear dominance between cells and bacteria. In terms of species-specific behaviour, *E. faecalis* was more sensitive to the Nano surface, while *S. oralis* more readily colonised SLA, confirming fundamental interspecies differences in colonisation strategies across various surface types [Piñera-Avellaneda et al., 2021; Gil & Sanz, 2025] [2,30].

Our results showed that both bacterial species were able to form biofilm on all three tested surface types, but in different amounts, suggesting distinct mechanisms of adaptation and colonisation. Notably, Nano-type implants exhibited the lowest levels of biofilm formation for both species, indicating that nanostructured topographies can limit biofilm stabilisation and maturation even under conditions favourable to contamination. In contrast, biofilm development was more pronounced on SLA and Ma surfaces, confirming previous reports that surface roughness and morphology directly influence both bacterial attachment and extracellular matrix formation [Robles et al., 2023; Camargo et al., 2020] [1,11]. Thus, the Nano surface was confirmed as the most favourable for host cells and the least permissive to bacteria; however, the bacterial response varied by species, underscoring the importance of microbial characteristics in the interaction with biomaterials.

### 4.2. Study Limitations

Although the results obtained provide valuable insights into the interaction between osteoblastic cells and bacteria on different implant surface types, several limitations should be acknowledged. Firstly, the experiments were conducted in vitro, with a limited sample size, during a short evaluation time, under controlled conditions, which do not fully reproduce the complexity of the oral environment, where factors such as saliva, mixed microbial flora, immune responses, and mechanical forces can significantly influence both bacterial and cellular behaviour. Secondly, only two bacterial species were analysed—selected for their clinical relevance—whereas, in reality, the peri-implant biofilm comprises a much more diverse microbial community with complex interspecies interactions. Moreover, the observation period was limited to a few days, which does not capture the long-term dynamic processes of biofilm maturation and bone remodelling. Finally, the use of a single cell line, the dental DF-derived cells, and no immune cells may not fully reflect the behaviour of mature osteoblasts in vivo. Also the co-culture conditions which were chosen so as to give the chance of survival for both pre-differentiated DF and bacterial strains. These limitations highlight the need for further studies, both in vitro, using more complex microbial models, and in vivo, to validate the clinical relevance of the present findings.

### 4.3. Future Perspectives

In vivo studies should be conducted, where the three types of models would be reproduced, in order to gather data in a more appropriate environment like the one of the human oral cavities.

## 5. Conclusions

Our study demonstrated that nanostructured surfaces provide an optimal balance between supporting cellular adhesion and hindering bacterial colonisation. The order of colonisation significantly influenced the competition for surface attachment, emphasising the importance of minimising intraoperative and postoperative contamination for the clinical success of dental implants. Moreover, the results indicate that Nano surfaces can reduce bacterial biofilm formation, thereby reducing the risk of peri-implant infections and contributing to more efficient long-term integration.

## Data Availability

The original contributions presented in this study are included in the article. Further inquiries can be directed to the corresponding author.
